# Association of Race, Socioeconomic Factors, and Treatment Characteristics With Overall Survival in Patients With Limited-Stage Small Cell Lung Cancer

**DOI:** 10.1001/jamanetworkopen.2020.32276

**Published:** 2021-01-12

**Authors:** Kexun Zhou, Huashan Shi, Ruqin Chen, Jordan J. Cochuyt, David O. Hodge, Rami Manochakian, Yujie Zhao, Sikander Ailawadhi, Yanyan Lou

**Affiliations:** 1 Division of Hematology and Medical Oncology, Mayo Clinic, Jacksonville, Florida; 2 Division of Biomedical Statistics and Informatics, Mayo Clinic, Jacksonville, Florida

## Abstract

**Importance:**

It has been established that disparities in race and socioeconomic status are associated with outcomes of non–small cell lung cancer. However, it remains unknown whether this extends to stage I, II, or III small cell lung cancer (SCLC), or limited-stage SCLC (L-SCLC).

**Objective:**

To investigate the associations of race, socioeconomic factors, and treatment characteristics with survival among patients with L-SCLC.

**Design, Setting, and Participants:**

Demographic information for patients with L-SCLC diagnosed between 2004 and 2014 was obtained from the National Cancer Database. The follow-up end point is death or last follow-up (date of last contact). Patients were divided into 5 mutually exclusive cohorts by race. Data analysis was performed in October 2019.

**Main Outcomes and Measures:**

Cox proportional hazards models were used to calculate univariable and multivariable models. Multivariable analyses were conducted to assess the associations of race and socioeconomic factors with risk-adjusted outcomes. Overall survival between groups was depicted by Kaplan-Meier curves.

**Results:**

Of 72 409 patients analyzed (median [range] age, 67.0 [23.0-90.0] years), 40 289 (55.6%) were women. The distribution of disease stage was 10 619 patients (14.7%) with stage I disease, 7689 patients (10.6%) with stage II disease, and 54 101 patients (74.7%) with stage III disease. The median (range) duration of follow-up was 8.2 (2.4-15.8) months. Compared with White patients, the hazard of death decreased to 0.92 (95% CI, 0.89-0.95; *P* < .001) for African American patients and 0.83 (95% CI, 0.77-0.91; *P* < .001) for Asian patients. The difference in median survival among different racial groups was significant only among those with stage III SCLC. Other factors associated with better survival were female sex, high income, high education, private insurance, diagnostic confirmation by positive cytological analysis, increase in number of sampled regional lymph nodes, and earlier stage at diagnosis.

**Conclusions and Relevance:**

This analysis highlights disparities in race and socioeconomic factors associated with outcomes of L-SCLC. Racial minorities, including African American and Asian patients, have better survival than White patients for L-SCLC after adjustment for sociodemographic factors.

## Key Points

**Question:**

Are disparities in race and socioeconomic status associated with outcomes among patients with limited-stage small cell lung cancer?

**Findings:**

In this cohort study including 72 409 patients, data from the National Cancer Database indicated that members of racial minorities, including African American and Asian patients, have better survival than White patients. Other factors associated with better survival were female sex, high income, high education, private insurance, diagnostic confirmation by positive cytological analysis, increase in number of sampled regional lymph nodes, and earlier stage at diagnosis.

**Meaning:**

These findings highlight the disparities in race and socioeconomic factors associated with outcomes of limited-stage small cell lung cancer.

## Teaser

This cohort study investigates the associations of race, socioeconomic factors, and treatment characteristics with overall survival (OS) among patients with limited-stage small cell lung cancer (SCLC).

## Introduction

Lung cancer is the leading cause of cancer-related mortality in the US, with 228 150 new cases and 142 670 deaths in 2019.^1^ Lung cancer can be broadly classified into 2 types: small cell lung cancer (SCLC) and non-SCLC (NSCLC).^2^ Although SCLC accounts for only approximately 14% of all lung cancers, it has inferior outcomes compared with NSCLC.^3^ As screening with low-dose computed tomography among high-risk populations is promoted, patients with lung cancer are more likely receive a diagnosis at limited stages when they could benefit from curative treatments, such as concurrent chemoradiation therapy.^4,5,6^

In addition to clinical characteristics and medical care, existing evidence indicates that economic status, medical insurance, sex, and age are associated with the occurrence and outcomes of cancer.^7,8,9^ Race is also considered a factor, primarily because of disparities in access to high-quality cancer prevention, early detection, and treatment^10,11^; however, this remains controversial for lung cancer. Some studies have attributed higher morbidity and mortality in African American patients to socioeconomic variables.^12,13,14^ Others have concluded that race is not an independent predictor of survival when controlling for confounding variables.^8,15,16^ Notably, most of these studies were limited by institutional experiences or statewide databases and mainly focused on NSCLC and extensive-stage SCLC.^17,18,19^ As a result, the validity of race, among other factors, for limited-stage (stages I-III) SCLC (L-SCLC) is not well understood. Unlike extensive-stage SCLC, which is incurable and primarily treated with systemic chemotherapy with or without immunotherapy, L-SCLC is potentially curable and treated primarily with concurrent chemoradiotherapy or surgery followed by adjuvant chemotherapy.^20^ We analyzed here the National Cancer Database (NCDB),^21^ which is one of the largest clinical cancer registries in the world and contains an array of medical and sociodemographic information on patients with cancer, to investigate the associations of race and socioeconomic factors with survival among patients with L-SCLC.

## Methods

The Mayo Clinic institutional review board gave prior approval for this cohort study using deidentified data and waived the need for written informed consent. The study is compliant with the Health Insurance Portability and Accountability Act and follows the Strengthening the Reporting of Observational Studies in Epidemiology (STROBE) reporting guideline.^22^

We identified patients who received a diagnosis of L-SCLC from 2004 to 2014. Patients were divided into 5 mutually exclusive cohorts by race/ethnicity (ie, Hispanic, Asian, African American, Native American, and White) as reported by patients in the NCDB. The residential region was consolidated into 3 categories: urban, metro, and rural. Median annual household income was categorized as less than $38 000, $38 000 to $47 999, $48 000 to $62 999, and $63 000 or more, as reported in the NCDB. Education level was grouped by the percentage of adults with no high school degree in the residential region: less than 7.0%, 7.0% to 12.9%, 13.0% to 20.9%, and 21.0% or greater. Insurance categories included no insurance, government insurance, and private insurance. Diagnostic confirmation was categorized as histological and cytological analyses, and facility type was classified as non–academic or research program and academic or research program. The Charlson-Deyo Comorbidity Index score was used for comorbidity status. Treatments were divided into surgery, radiotherapy, and chemotherapy.

### Statistical Analysis

Differences between races for categorical variables were analyzed by χ^2^ tests. Continuous variables were compared using the analysis of variance. Overall survival was calculated as the time from diagnosis to date of death or date last known alive (censored). The comparisons of the Kaplan-Meier curves between races for each stage were performed, and the log-rank test was used to compare differences between races in survival. Cox proportional hazards models were used to calculate univariable and multivariable models. Multivariable analysis was conducted to identify independent factors associated with the mortality among patients with L-SCLC, and hazard ratios (HRs) for mortality were reported. Proportional hazards assumptions were tested using Schoenfeld residuals, and nonproportional variables were used as stratification variables in the final models. Covariates used in this study included race/ethnicity, sex, type of living area, income, education, insurance status, diagnostic confirmation method, facility type, distance to treating facility, number of sampled regional lymph nodes, and cancer stages that were considered factors potentially associated with clinical outcomes. The primary analysis was the comparison of mortality between race groups. Secondary analyses were completed to assess the possible association of other factors with mortality. Because of the multiple comparisons, only *P* < .0125 was considered significant in the Cox proportional hazard model analysis. All statistical tests were 2-sided. Analysis was primarily completed in SAS statistical software version 9.4 (SAS Institute), and some graphics and tests used R-studio statistical software version 1.3 (R Project for Statistical Computing). Data analysis was performed in October 2019.

## Results

Of the 72 409 patients included (median [range] age, 67.0 [23.0-90.0] years), 40 289 (55.6%) were women and 32 120 (44.4%) were men. At diagnosis, 10 619 patients (14.7%) had stage I disease, 7689 (10.6%) had stage II disease, and 54 101 (74.7%) had stage III disease. The median (range) duration of follow-up was 8.2 (2.4-15.8) months. Patients’ demographic, clinical, and treatment features are summarized in Table 1. Stage-specific information by races is summarized in eTable 1, eTable 2, and eTable 3 in the Supplement. Among the studied racial groups, White was the largest racial group (65 449 patients [90.4%]), followed by African American (5724 patients [7.9%]), Asian (817 patients [1.1%]), Hispanic (217 patients [0.3%]), and Native American (202 patients [0.3%]) patients. The Hispanic and Asian patient groups had more men (120 [55%] and 521 [64%] men, respectively), whereas women constituted a larger proportion of patients among the White (36 610 women [56%]), African American (3160 women [55%]), and Native American groups (126 women [62%]). Most patients (40 851 patients [56.4%]) had a Charlson-Deyo Comorbidity Index score of 0. Only 1834 patients (2.6%) lived in rural areas. African American patients tended to have lower income and education levels than Asian and White patients. Specifically, in the low-income group (<$38 000), African American patients accounted for the largest racial group (372 patients [47.1%] with stage I disease, 298 patients [47.8%] with stage II disease, and 2146 patients [50.8%] with stage III disease). The same trend was observed in the lower education level group, in which African American patients accounted for 35.8% (283 patients) of those with stage I disease, 37.2% (232 patients) of those with stage II disease, and 38.7% (1634 patients) of those with stage III disease. More than 90% of studied patients across all races (68 769 patients) had some form of medical insurance coverage and most had government insurance (7660 patients [75%] with stage I disease, 5227 patients [71%] with stage II disease, and 35 725 patients [70%] with stage III disease). Eighty-three percent of patients (59 962 patients) were diagnosed according to positive histological findings, and 74% of patients (53 551 patients) were treated at non–academic or research programs across all races. The exception to this was noted when patients were analyzed by stage, showing that 65% of Hispanic patients (26 patients) with stage I SCLC were treated at academic or research programs. However, the overall number of Hispanic patients remained low.

**Table 1.** Patients’ Demographic, Clinical, and Treatment Characteristics

Characteristic	Patients, No. (%) (N = 72 409)
Cancer stage	
I	10 619 (14.7)
II	7689 (10.6)
III	54 101 (74.7)
Race/ethnicity	
White	65 449 (90.4)
African American	5724 (7.9)
Asia,	817 (1.1)
Native American	202 (0.3)
Hispanic	217 (0.3)
Sex	
Male	32 120 (44.4)
Female	40 289 (55.6)
Age group, y	
<60	17 354 (24.0)
60-69	24 637 (34.0)
70-79	22 272 (30.8)
≥80	8146 (11.2)
Age at diagnosis, y	
Mean (SD)	67.0 (10.2)
Median (range)	67.0 (23.0-90.0)
Type of area	
Missing^a^	2644
Urban	13 063 (18.7)
Metro	54 868 (78.6)
Rural	1834 (2.6)
US Census annual household median income, $^b^	
Missing^a^	1412
<38 000	15 555 (21.9)
38 000-47 999	19 705 (27.8)
48 000-62 999	19 034 (26.8)
≥63 000	16 703 (23.5)
No high school degree, %^b^	
Missing^a^	1372
≥21	13 409 (18.9)
13-20.9	21 674 (30.5)
7-12.9	23 639 (33.3)
<7	12 315 (17.3)
Insurance	
Missing^a^	2343
None	1297 (1.9)
Government	48 612 (69.4)
Private	20 157 (28.8)
Year of diagnosis	
2004	6434 (8.9)
2005	6474 (8.9)
2006	6332 (8.7)
2007	6238 (8.6)
2008	6924 (9.6)
2009	7027 (9.7)
2010	6564 (9.1)
2011	6405 (8.8)
2012	6632 (9.2)
2013	6682 (9.2)
2014	6697 (9.2)
Diagnostic confirmation	
Missing^a^	36
Positive histological findings	59 962 (82.9)
Positive cytological findings	12 411 (17.1)
Facility type	
Missing^a^	229
Non–academic or research program	53 551 (74.2)
Academic or research program	18 629 (25.8)
Distance to treating facility, miles	
Mean (SD)	23.5 (83.6)
Median (range)	9.2 (0.0-4786.9)
Lymph nodes^c^	
Mean (SD)	13.4 (32.4)
Median (range)	0.0 (0.0-99.0)
Charlson-Deyo Comorbidity Index score	
0	40 851 (56.4)
1	21 650 (29.9)
2	7460 (10.3)
≥3	2448 (3.4)
Surgery of primary site	
Missing^a^	223
No	66 692 (92.4)
Yes	5494 (7.6)
Radiation therapy	
Missing^a^	444
No	26 148 (36.3)
Yes	45 817 (63.7)
Chemotherapy	
Missing^a^	1174
No	13 337 (18.7)
Yes	57 898 (81.3)

SI conversion factor: To convert miles to kilometers, multiply by 1.60934.

^a^ Missing data were not included in calculating the percentages.

^b^ Variables refer to the residential region, rather than individual.

^c^ Refers to regional lymph nodes.

With regard to tumor and treatment characteristics, although the overall distribution was consistent, some differences were noticed. Among those with stage I SCLC, African American patients had lower rates of undergoing surgery for the primary site (eTable 1 in the Supplement), whereas there was no significant difference among races in patients with stage II or III disease (eTables 2 and 3 in the Supplement). There was a difference in regional lymph nodes sampled among racial groups in patients with stage I and III SCLC (eTables 1 and 3 in the Supplement). No difference was found in the administration of radiation therapy among racial subgroups for any of the disease stages. Chemotherapy was administered in most patients, and the proportion of patients was not significantly different among the racial groups, except for stage II, where more Hispanic patients received chemotherapy than White, African American, Asian, and Native American patients (eTable 2 in the Supplement).

Median survival estimated by stage across different races is shown in eTable 1, eTable 2, and eTable 3 in the Supplement, and Kaplan-Meier curves are depicted in the Figure. Median survival ranged from 27.7 months (95% CI, 19.4 months to not reached) in Hispanic patients to 22.0 months (95% CI, 21.3-22.7 months) in White patients, with no significant difference across races in patients with stage I SCLC (FigureA; *P* = .54). Similarly, for stage II disease, the median survival ranged from 20.4 months (95% CI, 15.0-28.0 months) in Asian patients to 17.5 months (95% CI, 14.8 months to not reached) in Hispanic patients, with no significant difference across races (FigureB; *P* = .70). Among patients with stage III SCLC, survival was significantly different among races, with median survival of 12.7 months (95% CI, 12.6-12.9 months) in White patients compared with 13.6 months (95% CI, 13.2-14.1 months) in African American patients, 13.4 months (95% CI, 10.3-16.9 months) in Hispanic patients, 13.9 months (95% CI, 12.6-15.2 months) in Asian patients, and 13.5 months (95% CI, 11.3-16.7 months) in Native American patients (Figure panel C; *P* < .001). Multivariable analysis revealed that race/ethnicity, sex, income, education, type of insurance, diagnostic confirmation, number of sampled regional lymph nodes, and disease stage were significantly associated with the survival of patient (Table 2). African American (HR, 0.92; 95% CI, 0.89-0.95; *P* < .001) and Asian (HR, 0.83; 95% CI, 0.77-0.91; *P* < .001) patients exhibited a lower likelihood of dying than White patients. Female sex (HR, 0.84; 95% CI, 0.83-0.85, *P* < .001), annual median income greater than or equal to $63 000 (HR, 0.94; 95% CI, 0.90-0.98, *P* < .001), high education (HR, 0.94; 95% CI, 0.91-0.98, *P* = .002), private insurance (HR, 0.88; 95% CI, 0.81-0.94, *P* < .001), diagnosis confirmation by positive cytological findings (HR, 0.96; 95% CI, 0.94-0.98; *P* = .003), increase in number of sampled regional lymph nodes (HR, 0.99; 95% CI, 0.98-0.99, *P* < .001), and earlier stage at diagnosis were associated with longer survival.

**Figure.**
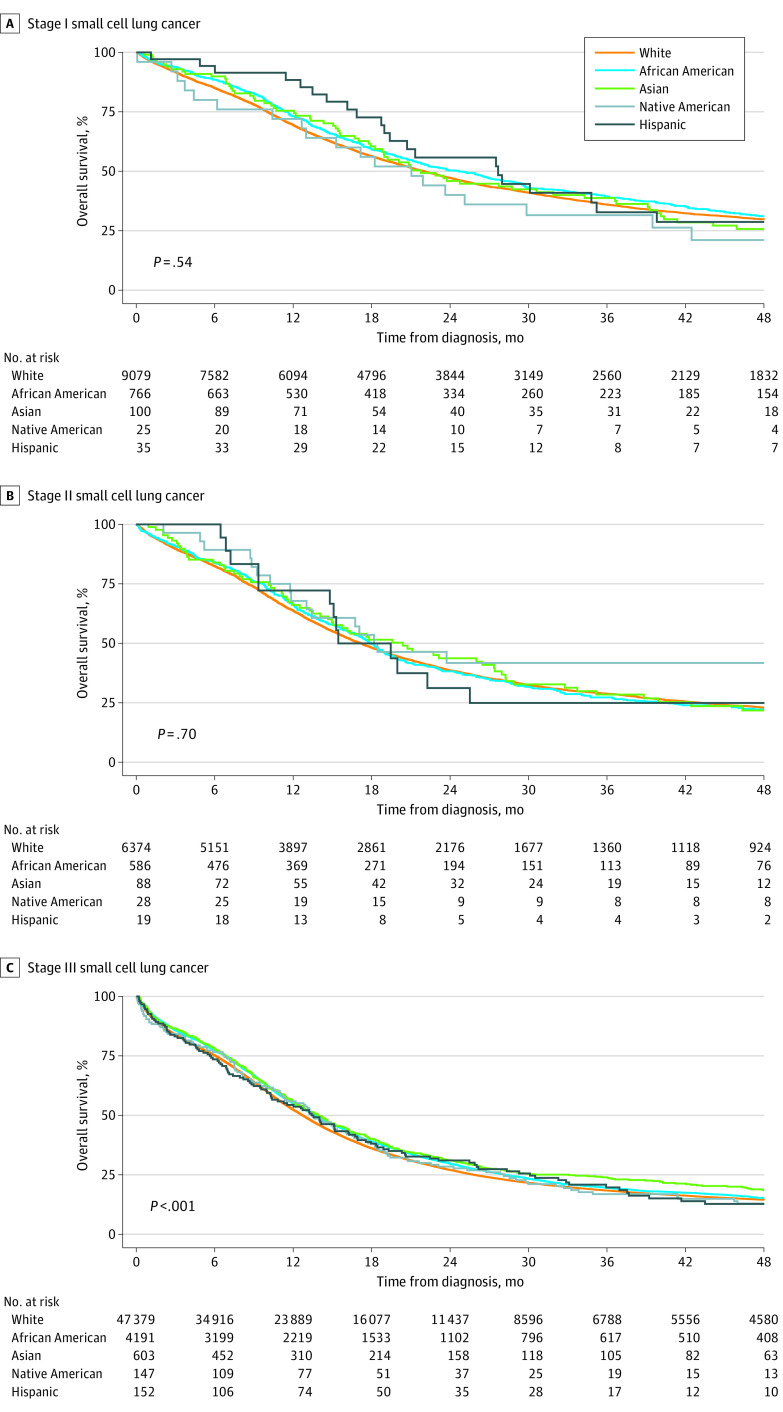
Survival Among Patients Stratified by Race/Ethnicity Graphs show Kaplan-Meier survival curves for patients with stage I (A), stage II (B), and stage III (C) small cell lung cancer.

**Table 2.** Results From Cox Proportional Hazard Model for Mortality

Variable	Hazard ratio (95% CI)	*P* value
Race/ethnicity		
White	1.00 [Reference]	
Hispanic	1.03 (0.87-1.22)	.76
Asian	0.83 (0.77-0.91)	<.001
African American	0.92 (0.89-0.95)	<.001
Native American	0.95 (0.82-1.13)	.62
Sex		
Male	1.00 [Reference]	
Female	0.84 (0.83-0.85)	<.001
Type of area		
Urban	1.00 [Reference]	
Metro	1.02 (0.99-1.04)	.11
Rural	1.07 (1.01-1.13)	.02
US Census annual household median income, $^a^		
<38 000	1.00 [Reference]	
38 000-47 999	0.98 (0.95-1.01)	.13
48 000-62 999	0.98 (0.95-1.01)	.15
≥63 000	0.94 (0.90-0.98)	<.001
No high school degree, %^a^		
≥21	1.00 [Reference]	
13.0-20.9	1.01 (0.99-1.04)	.35
7.0-12.9	0.98 (0.95-1.01)	.22
<7.0	0.94 (0.91-0.98)	.002
Insurance		
None	1.00 [Reference]	
Government	1.01 (0.95-1.09)	.61
Private	0.88 (0.81-0.94)	<.001
Diagnostic confirmation		
Positive histological findings	1.00 [Reference]	
Positive cytological findings	0.96 (0.94-0.98)	.003
Facility type		
Non–academic or research program	1.00 [Reference]	
Academic or research program	0.98 (0.96-1.00)	.05
Distance to treating facility (50-unit increase)	0.99 (0.98-0.999)	.02
Lymph nodes (10-unit increase)^b^	0.99 (0.98-0.99)	<.001
Cancer stage		
I	1.00 [Reference]	
II	1.27 (1.22-1.32)	<.001
III	1.78 (1.73-1.83)	<.001

^a^ Variables refer to the residential region, rather than individual.

^b^ Refers to regional lymph nodes.

## Discussion

Breakthroughs in early detection and treatment of lung cancer have improved clinical outcomes in the last decade.^1^ However, this improvement has not been universal across all racial and socioeconomic groups. Because the literature is divided and there is a lack of large-scale studies in L-SCLC, we used the NCDB to examine disparities in the survival outcome of L-SCLC according to race, socioeconomic indicators, and treatment modalities.

It has been frequently reported that White patients have better outcomes, whereas African American patients are more likely to receive a diagnosis of cancer at an advanced stage and have higher mortality rates than other racial groups.^1,22,23^ Using the Southwest Oncology Group database, Albain et al^24^ analyzed 2580 patients with SCLC. As the Cox multivariable analyses revealed, White patients with limited-stage disease had improved survival compared with other racial and ethnic groups (*P* < .04).^24^ LaPar et al,^9^ using the Nationwide Inpatient Sample database, identified 129 207 patients with lung cancer who underwent surgery from 2002 to 2007. Their results indicated that African American patients had a lower incidence of adverse effects and mortality than White patients.^9^ Another meta-analysis^25^ aiming to study the association of race with the survival of patients with lung cancer suggested that when clinical factors and smoking status were adjusted for, Asian and Hispanic patients experienced improved survival compared with non-Hispanic White patients. In contrast, no significant difference in survival was found between African American and White patients after adjustment.^25^ However, our analysis indicated that African American and Asian patients had significantly decreased mortality rates compared with White patients. The results are supported by the findings of Fillmore et al^26^; using data from Veterans Affairs hospitals, they found that African American patients with multiple myelomas had better overall survival than White patients. In addition, using different databases, variables, and analyses may yield inconsistent results. Most published studies are either focused on lung cancer in general, NSCLC, or SCLC and use a smaller data set. Our research primarily focused on L-SCLC, rather than lung cancer in general or NSCLC, and included a large number of patients managed in more recent years with updated analysis. Furthermore, disease biology can vary in different histological types of lung cancer, contributing to inconsistent conclusions. Further studies to validate our findings are warranted.

The risk of death varied by sex, as men in our study had worse outcomes than women. This finding is consistent with those of several previous publications. Wang et al^27^ used the National Cancer Institute Surveillance Epidemiology and End Results database to evaluate the survival change among 56 220 patients with SCLC and observed that women had lower mortality rates. Another retrospective study^28^ of the NCDB also indicated that the risk of death decreased among women with early-stage SCLC who underwent surgery of the primary site.

In terms of socioeconomic factors, our analysis was largely in agreement with published studies. High income status significantly reduced the hazard of death, which is consistent with findings from a study of NSCLC conducted by Ou et al,^29^ who analyzed 19 702 patients with stage I NSCLC and found that improvement in socioeconomic status significantly decreased HRs for death.^29^ Another study^30^ assessing the correlation between social factors and survival among patients with early-stage NSCLC drew the same conclusion.

Survival advantage was observed for patients with higher education levels in our study. Di Maio et al^31^ investigated the association of education level with survival in patients with advanced NSCLC and observed that patients with higher education level had longer overall survival, but their findings remained controversial. Herndon et al^32^ investigated the association of education with survival among patients with lung cancer and reported that after being enrolled in a clinical trial, the survival of patients with SCLC was not associated with education, even though the physical conditions of candidates with lower education levels were worse. Besides, a meta-analysis by Finke et al^33^ did not confirm associations between individual education and clinical outcomes. This should be verified by further study.

According to our analysis, type of insurance was also associated with survival, as patients covered by private insurance had lower risk of death, consistent with another NCDB analysis by Pezzi et al,^34^ who examined whether Medicaid coverage was associated with survival among patients with SCLC. Consistent with our findings, private and Medicare insurance were found to be associated with survival benefit in patients with SCLC, regardless of stage, although Medicaid was not associated with improved survival.^34^ Similarly, MacLean et al^35^ obtained data from 2006 to 2012 to compare the application of neoadjuvant and adjuvant chemotherapy in stage II and III NSCLC. Private insurance was shown to provide more survival advantage compared with public insurance.^35^ Biswas et al^8^ suggested that different insurance status could also contribute to disparities in overall survival of patients with early-stage NSCLC. Patients with private insurance had better outcomes than those with other insurance types.^8^ Groth et al^36^ suggested that patients with private insurance were more likely to be offered curative surgery.

With regard to facility types, although there is no specific study of this issue in L-SCLC, previous studies^37,38,39^ indicated that patients with NSCLC, oral cancer, or pancreatic adenocarcinoma treated at academic centers were more likely to have more prolonged survival. Our finding supports this conclusion in L-SCLC.

In our study, we found that an increased number of sampled regional lymph nodes was associated with decreased hazard of death. It has been well established that outcomes are strongly associated with lymph node resection. Watanabe et al^40^ highlighted the significance of lymph node dissection in lung cancer, which allows for more accurate pathological staging and more proper treatment. By analyzing the Surveillance Epidemiology and End Results database, Yendamuri et al^41^ found that more extensive lymph node resection was associated with an increased survival benefit for patients with early stage NSCLC.

### Limitations

To our knowledge, our study is the first large-scale database study analyzing various factors associated with outcomes of L-SCLC; however, it is not without limitations. The median (range) follow-up is 8.2 (2.4-15.8) months, which is short and potentially flawed by selection bias due to small case numbers. Second, selection bias should be considered in retrospective studies, and caution should be exercised in interpreting the results with different sample sizes. In our study, patients with stage III L-SCLC accounted for 74.7% of the total studied population. In contrast, the numbers of patients with stage I and II L-SCLC and patients in the Hispanic and Native American racial groups were small, which might have affected the statistical power. In addition, different access to screening programs among racial groups may lead to diagnosis at an earlier stage of SCLC in some racial groups than others. This might result in a potential selection bias. Previous studies mainly focused on the majority population (White and African American patients). However, the size of minorities has grown over time in the US, and it is reasonable to take these patient populations into consideration.^42^ Furthermore, patients enrolled in our study received a diagnosis of L-SCLC from 2004 to 2014. Progress in treating L-SCLC has been witnessed after this study period. For example, adjuvant chemotherapy with or without radiation therapy is associated with improved overall survival in T1-2N0M0 SCLC after complete surgical resection.^6^ Twice-daily thoracic radiation therapy concurrent with cisplatin and etoposide is reemphasized as the standard of care for limited-stage SCLC according to the CONVERT trial.^43^ These advances may improve the outcome of L-SCLC but are likely missing from our study because of data availability. Although the NCDB contains comprehensive information on cancer care in the US, some individual patient information is not available. For example, individual comorbidity and details of treatment are not specified. Also, as multimodality therapy is emphasized in the treatment of L-SCLC, data obtained from the NCDB cannot consistently distinguish concurrent from sequential chemotherapy and radiotherapy.^21^ Furthermore, treatments are within-person, time-dependent variables, and there is a possibility of introducing immortal time bias. This omitted information could have affected the analysis of outcomes in L-SCLC.

## Conclusions

The findings of this cohort study suggest that race is associated with survival of L-SCLC, because Asian and African American patients had better survival compared with White patients. In addition, our results confirmed that female sex, higher median annual income (≥$63 000), private insurance, diagnosis confirmation by positive cytological analysis, larger increase in number of sampled regional lymph nodes examined, and earlier stage at diagnosis were associated with prolonged survival in L-SCLC. Given that there is a complex interaction among several factors, more studies are warranted to formulate strategies to overcome the disparities that we have highlighted.

## Notes

Supplement. **eTable 1. **Baseline Characteristics and Treatments of Patients With Stage I SCLC **eTable 2. ** Baseline Characteristics and Treatments of Patients With Stage II SCLC **eTable 3.** Baseline Characteristics and Treatments of Patients With Stage III SCLC
