# Supplementary material for: Association of Race, Socioeconomic Factors, and Treatment Characteristics With Overall Survival in Patients With Limited-Stage Small Cell Lung Cancer
Source: JAMA Netw Open. 2021 Jan 12;4(1):e2032276. doi: 10.1001/jamanetworkopen.2020.32276 (PMC7804918; doi:10.1001/jamanetworkopen.2020.32276)
Supplement: Supplement. — eTable 1. Baseline Characteristics and Treatments of Patients With Stage I SCLC eTable 2. Baseline Characteristics and Treatments of Patients With Stage II SCLC eTable 3. Baseline Characteristics and Treatments of Patients With Stage III SCLC [file jamanetwopen-e2032276-s001.pdf]

Supplemental Online Content

Zhou K, Shi H, Chen R, et al. Association of race, socioeconomic factors, and treatment characteristics with overall survival in patients with limited-stage small cell lung cancer. *JAMA Netw Open*. 2021;4(1):e2032276. doi:10.1001/jamanetworkopen.2020.32276

eTable 1. Baseline Characteristics and Treatments of Patients With Stage I SCLC

eTable 2. Baseline Characteristics and Treatments of Patients With Stage II SCLC

eTable 3. Baseline Characteristics and Treatments of Patients With Stage III SCLC

This supplemental material has been provided by the authors to give readers additional information about their work.

© 2021 Zhou K et al. *JAMA Network Open.*

eTable 1. **Baseline Characteristics and Treatments of Patients With Stage I SCLC**

	Hispanic (N=40) [a]	Asian (N=110) [a]	AA (N=798) [a]	NA (N=26) [a]	Caucasian (N=9645) [a]	p value
**Sex**						<.001
Male	23 (57.5)	79 (71.8)	360 (45.1)	11 (42.3)	4325 (44.8)	
Female	17 (42.5)	31 (28.2)	438 (54.9)	15 (57.7)	5320 (55.2)	
**Age group**						<.001
<60	8 (20.0)	17 (15.5)	206 (25.8)	8 (30.8)	1556 (16.1)	
60-69	10 (25.0)	33 (30.0)	249 (31.2)	12 (46.2)	3097 (32.1)	
70-79	17 (42.5)	38 (34.5)	246 (30.8)	5 (19.2)	3588 (37.2)	
>=80	5 (12.5)	22 (20.0)	97 (12.2)	1 (3.8)	1404 (14.6)	
**Type of area**						<.001
Missing	1	3	20	1	342	
Urban	4 (10.3)	4 (3.7)	65 (8.4)	13 (52.0)	1814 (19.5)	
Metro	35 (89.7)	103 (96.3)	709 (91.1)	8 (32.0)	7236 (77.8)	
Rural	0 (0.0)	0 (0.0)	4 (0.5)	4 (16.0)	253 (2.7)	
**Census Median Income** [b]						<.001
Missing	1	0	8	0	176	
<$38,000	10 (25.6)	7 (6.4)	372 (47.1)	8 (30.8)	1793 (18.9)	
$38,000-$47,999	9 (23.1)	19 (17.3)	176 (22.3)	12 (46.2)	2626 (27.7)	
$48,000-$62,999	11 (28.2)	35 (31.8)	154 (19.5)	5 (19.2)	2675 (28.3)	
$63,000+	9 (23.1)	49 (44.5)	88 (11.1)	1 (3.8)	2375 (25.1)	
**No High School Degree** [c]						<.001
Missing	1	0	8	0	170	
>=21	15 (38.5)	18 (16.4)	283 (35.8)	4 (15.4)	1524 (16.1)	
13-20.9	13 (33.3)	30 (27.3)	274 (34.7)	10 (38.5)	2866 (30.2)	
7-12.9	6 (15.4)	28 (25.5)	162 (20.5)	9 (34.6)	3267 (34.5)	
<7	5 (12.8)	34 (30.9)	71 (9.0)	3 (11.5)	1818 (19.2)	
**Insurance**						.06
Missing	0	3	24	0	193	
No insurance	3 (7.5)	0 (0.0)	19 (2.5)	0 (0.0)	169 (1.8)	
Government Insurance	30 (75.0)	76 (71.0)	577 (74.5)	22 (84.6)	6955 (73.6)	
Private insurance	7 (17.5)	31 (29.0)	178 (23.0)	4 (15.4)	2328 (24.6)	
**Year of Diagnosis**						.52
2004	4 (10.0)	10 (9.1)	70 (8.8)	4 (15.4)	917 (9.5)	
2005	4 (10.0)	17 (15.5)	64 (8.0)	1 (3.8)	903 (9.4)	
2006	2 (5.0)	7 (6.4)	64 (8.0)	2 (7.7)	831 (8.6)	
2007	4 (10.0)	8 (7.3)	76 (9.5)	3 (11.5)	873 (9.1)	
2008	2 (5.0)	15 (13.6)	66 (8.3)	4 (15.4)	1001 (10.4)	
2009	4 (10.0)	8 (7.3)	70 (8.8)	3 (11.5)	956 (9.9)	
2010	3 (7.5)	10 (9.1)	76 (9.5)	4 (15.4)	849 (8.8)	
2011	2 (5.0)	7 (6.4)	67 (8.4)	3 (11.5)	799 (8.3)	

© 2021 Zhou K et al. *JAMA Network Open.*

2012	6 (15.0)	5 (4.5)	89 (11.2)	1 (3.8)	829 (8.6)	
2013	4 (10.0)	12 (10.9)	73 (9.1)	0 (0.0)	851 (8.8)	
2014	5 (12.5)	11 (10.0)	83 (10.4)	1 (3.8)	836 (8.7)	
**Diagnostic confirmation**						.12
Missing	0	0	1	0	4	
Positive histology	36 (90.0)	93 (84.5)	674 (84.6)	26 (100.0)	8341 (86.5)	
Positive cytology	4 (10.0)	17 (15.5)	123 (15.4)	0 (0.0)	1300 (13.5)	
**Facility Type**						<.001
Missing	0	0	3	0	21	
Non-A/R Program	14 (35.0)	66 (60.0)	492 (61.9)	20 (76.9)	7143 (74.2)	
A/R Program	26 (65.0)	44 (40.0)	303 (38.1)	6 (23.1)	2481 (25.8)	
**Distance to treating facility, mean (SD), mile**	12.1 (14.5)	12.6 (24.1)	11.7 (27.4)	44.3 (37.2)	24.9 (85.0)	<.001
**Lymph nodes, mean (SD) [d]**	12.4 (29.7)	6.7 (20.5)	6.7 (22.4)	2.5 (4.3)	7.7 (23.3)	.008
**Charlson-Deyo Score**						.049
0	27 (67.5)	73 (66.4)	461 (57.8)	14 (53.8)	5342 (55.4)	
1	6 (15.0)	27 (24.5)	221 (27.7)	6 (23.1)	2964 (30.7)	
2	5 (12.5)	5 (4.5)	86 (10.8)	6 (23.1)	1021 (10.6)	
>=3	2 (5.0)	5 (4.5)	30 (3.8)	0 (0.0)	318 (3.3)	
**Surgery of Primary Site**						.003
Missing	1	0	3	0	38	
No	27 (69.2)	76 (69.1)	619 (77.9)	18 (69.2)	6859 (71.4)	
Yes	12 (30.8)	34 (30.9)	176 (22.1)	8 (30.8)	2748 (28.6)	
**Radiation Therapy**						.40
Missing	1	2	7	0	67	
No	19 (48.7)	47 (43.5)	344 (43.5)	13 (50.0)	4494 (46.9)	
Yes	20 (51.3)	61 (56.5)	447 (56.5)	13 (50.0)	5084 (53.1)	
**Chemotherapy**						.38
Missing	1	7	22	0	216	
No	7 (17.9)	33 (32.0)	229 (29.5)	5 (19.2)	2693 (28.6)	
Yes	32 (82.1)	70 (68.0)	547 (70.5)	21 (80.8)	6736 (71.4)	
**Median Survival (95CI), mo**	27.7 (19.4-NA)	21.8 (18.8-36.6)	24.7 (21.8-27.9)	21.0 (13.0-42.5)	22. 0 (21.3-22.7)	.54

© 2021 Zhou K et al. *JAMA Network Open.*

eTable 2. Baseline Characteristics and Treatments of Patients With Stage II SCLC

	Hispanic (N=21) [a]	Asian (N=90) [a]	AA (N=631) [a]	NA (N=29) [a]	Caucasian (N=6918) [a]	p value
**Sex**						.006
Male	14 (66.7)	55 (61.1)	290 (46.0)	10 (34.5)	3129 (45.2)	
Female	7 (33.3)	35 (38.9)	341 (54.0)	19 (65.5)	3789 (54.8)	
**Age group**						<.001
<60	5 (23.8)	6 (6.7)	174 (27.6)	8 (27.6)	1517 (21.9)	
60-69	6 (28.6)	30 (33.3)	212 (33.6)	16 (55.2)	2327 (33.6)	
70-79	9 (42.9)	34 (37.8)	190 (30.1)	1 (3.4)	2264 (32.7)	
>=80	1 (4.8)	20 (22.2)	55 (8.7)	4 (13.8)	810 (11.7)	
**Type of area**						<.001
Missing	1	3	16	0	269	
Urban	2 (10.0)	3 (3.4)	61 (9.9)	9 (31.0)	1277 (19.2)	
Metro	18 (90.0)	84 (96.6)	549 (89.3)	16 (55.2)	5188 (78.0)	
Rural	0 (0.0)	0 (0.0)	5 (0.8)	4 (13.8)	184 (2.8)	
**Census Median Income [b]**						<.001
Missing	0	1	8	0	126	
<$38,000	5 (23.8)	7 (7.9)	298 (47.8)	7 (24.1)	1251 (18.4)	
$38,000-$47,999	4 (19.0)	13 (14.6)	150 (24.1)	10 (34.5)	1946 (28.7)	
$48,000-$62,999	3 (14.3)	26 (29.2)	113 (18.1)	6 (20.7)	1934 (28.5)	
$63,000+	9 (42.9)	43 (48.3)	62 (10.0)	6 (20.7)	1661 (24.5)	
**No High School Degree [c]**						<.001
Missing	0	1	8	0	126	
>=21	5 (23.8)	11 (12.4)	232 (37.2)	3 (10.3)	1101 (16.2)	
13-20.9	5 (23.8)	20 (22.5)	219 (35.2)	10 (34.5)	2092 (30.8)	
7-12.9	5 (23.8)	37 (41.6)	132 (21.2)	14 (48.3)	2345 (34.5)	
<7	6 (28.6)	21 (23.6)	40 (6.4)	2 (6.9)	1254 (18.5)	
**Insurance**						.85
Missing	0	4	17	0	186	
No insurance	1 (4.8)	0 (0.0)	12 (2.0)	1 (3.4)	154 (2.3)	
Government Insurance	16 (76.2)	62 (72.1)	433 (70.5)	19 (65.5)	4697 (69.8)	
Private insurance	4 (19.0)	24 (27.9)	169 (27.5)	9 (31.0)	1881 (27.9)	
**Year of Diagnosis**						.10
2004	2 (9.5)	2 (2.2)	48 (7.6)	0 (0.0)	582 (8.4)	
2005	2 (9.5)	7 (7.8)	44 (7.0)	1 (3.4)	564 (8.2)	
2006	2 (9.5)	6 (6.7)	37 (5.9)	3 (10.3)	561 (8.1)	
2007	1 (4.8)	14 (15.6)	43 (6.8)	5 (17.2)	499 (7.2)	
2008	2 (9.5)	6 (6.7)	49 (7.8)	3 (10.3)	540 (7.8)	
2009	2 (9.5)	8 (8.9)	44 (7.0)	1 (3.4)	518 (7.5)	
2010	1 (4.8)	7 (7.8)	69 (10.9)	4 (13.8)	723 (10.5)	
2011	2 (9.5)	6 (6.7)	75 (11.9)	5 (17.2)	682 (9.9)	

© 2021 Zhou K et al. *JAMA Network Open.*

2012	5 (23.8)	7 (7.8)	74 (11.7)	0 (0.0)	702 (10.1)	
2013	0 (0.0)	17 (18.9)	78 (12.4)	3 (10.3)	750 (10.8)	
2014	2 (9.5)	10 (11.1)	70 (11.1)	4 (13.8)	797 (11.5)	
**Diagnostic confirmation**						.35
Missing	0	1	0	0	3	
Positive histology	16 (76.2)	75 (84.3)	528 (83.7)	28 (96.6)	5790 (83.7)	
Positive cytology	5 (23.8)	14 (15.7)	103 (16.3)	1 (3.4)	1125 (16.3)	
**Facility Type**						<.001
Missing	0	0	4	0	16	
Non-A/R Program	13 (61.9)	53 (58.9)	398 (63.5)	27 (93.1)	5151 (74.6)	
A/R Program	8 (38.1)	37 (41.1)	229 (36.5)	2 (6.9)	1751 (25.4)	
**Distance to treating facility, mean (SD), mile**	17.5 (18.4)	12.7 (29.2)	14.4 (60.1)	45.6 (65.6)	24.1 (67.3)	<.001
**Lymph nodes, mean (SD) [d]**	9.8 (29.5)	8.5 (26.0)	8.6 (25.8)	16.4 (36.7)	11.6 (29.5)	.12
**Charlson-Deyo Score**						.005
0	16 (76.2)	56 (62.2)	343 (54.4)	18 (62.1)	3936 (56.9)	
1	1 (4.8)	18 (20.0)	196 (31.1)	10 (34.5)	2101 (30.4)	
2	4 (19.0)	10 (11.1)	57 (9.0)	1 (3.4)	663 (9.6)	
>=3	0 (0.0)	6 (6.7)	35 (5.5)	0 (0.0)	218 (3.2)	
**Surgery of Primary Site**						.21
Missing	0	0	1	0	23	
No	20 (95.2)	81 (90.0)	542 (86.0)	28 (96.6)	5901 (85.6)	
Yes	1 (4.8)	9 (10.0)	88 (14.0)	1 (3.4)	994 (14.4)	
**Radiation Therapy**						.92
Missing	0	1	2	0	49	
No	6 (28.6)	33 (37.1)	215 (34.2)	9 (31.0)	2403 (35.0)	
Yes	15 (71.4)	56 (62.9)	414 (65.8)	20 (69.0)	4466 (65.0)	
**Chemotherapy**						.03
Missing	0	1	11	0	124	
No	2 (9.5)	26 (29.2)	123 (19.8)	8 (27.6)	1231 (18.1)	
Yes	19 (90.5)	63 (70.8)	497 (80.2)	21 (72.4)	5563 (81.9)	
**Median Survival (95CI), mo**	17.5 (47.8-NA)	20.4 (15.0-28.0)	18.0 (16.7-19.1)	18.3 (13.0-NA)	17.2 (16.6-17.8)	.70

© 2021 Zhou K et al. *JAMA Network Open.*

eTable 3. Baseline Characteristics and Treatments of Patients With Stage III SCLC

	Hispanic (N=156) [a]	Asian (N=617) [a]	AA (N=4295) [a]	NA (N=147) [a]	Caucasian (N=48886) [a]	p value
**Sex**						<.001
Male	83 (53.2)	387 (62.7)	1914 (44.6)	55 (37.4)	21385 (43.7)	
Female	73 (46.8)	230 (37.3)	2381 (55.4)	92 (62.6)	27501 (56.3)	
**Age group**						<.001
<60	46 (29.5)	141 (22.9)	1343 (31.3)	41 (27.9)	12278 (25.1)	
60-69	47 (30.1)	202 (32.7)	1501 (34.9)	51 (34.7)	16844 (34.5)	
70-79	49 (31.4)	193 (31.3)	1086 (25.3)	47 (32.0)	14505 (29.7)	
>=80	14 (9.0)	81 (13.1)	365 (8.5)	8 (5.4)	5259 (10.8)	
**Type of area**						<.001
Missing	6	25	117	2	1838	
Urban	12 (8.0)	28 (4.7)	389 (9.3)	60 (41.4)	9322 (19.8)	
Metro	138 (92.0)	564 (95.3)	3731 (89.3)	60 (41.4)	36429 (77.4)	
Rural	0 (0.0)	0 (0.0)	58 (1.4)	25 (17.2)	1297 (2.8)	
**Census Median Income [b]**						<.001
Missing	3	11	73	2	1003	
<$38,000	34 (22.2)	63 (10.4)	2146 (50.8)	66 (45.5)	9488 (19.8)	
$38,000-$47,999	35 (22.9)	105 (17.3)	986 (23.4)	38 (26.2)	13576 (28.4)	
$48,000-$62,999	45 (29.4)	178 (29.4)	655 (15.5)	29 (20.0)	13165 (27.5)	
$63,000+	39 (25.5)	260 (42.9)	435 (10.3)	12 (8.3)	11654 (24.3)	
**No High School Degree [c]**						<.001
Missing	3	11	72	2	970	
>=21	42 (27.5)	157 (25.9)	1634 (38.7)	37 (25.5)	8343 (17.4)	
13-20.9	48 (31.4)	135 (22.3)	1597 (37.8)	50 (34.5)	14305 (29.9)	
7-12.9	43 (28.1)	206 (34.0)	771 (18.3)	40 (27.6)	16574 (34.6)	
<7	20 (13.1)	108 (17.8)	221 (5.2)	18 (12.4)	8694 (18.1)	
**Insurance**						<.001
Missing	11	29	231	5	1640	
No insurance	4 (2.8)	14 (2.4)	92 (2.3)	1 (0.7)	827 (1.8)	
Government Insurance	98 (67.6)	410 (69.7)	2983 (73.4)	120 (84.5)	32114 (68.0)	
Private insurance	43 (29.7)	164 (27.9)	989 (24.3)	21 (14.8)	14305 (30.3)	
**Year of Diagnosis**						.004
2004	14 (9.0)	41 (6.6)	347 (8.1)	9 (6.1)	4384 (9.0)	
2005	12 (7.7)	48 (7.8)	372 (8.7)	6 (4.1)	4429 (9.1)	
2006	10 (6.4)	32 (5.2)	382 (8.9)	16 (10.9)	4377 (9.0)	
2007	11 (7.1)	57 (9.2)	361 (8.4)	11 (7.5)	4272 (8.7)	
2008	11 (7.1)	57 (9.2)	378 (8.8)	19 (12.9)	4771 (9.8)	
2009	13 (8.3)	90 (14.6)	447 (10.4)	19 (12.9)	4844 (9.9)	
2010	15 (9.6)	52 (8.4)	414 (9.6)	12 (8.2)	4325 (8.8)	
2011	15 (9.6)	56 (9.1)	373 (8.7)	16 (10.9)	4297 (8.8)	
2012	21 (13.5)	66 (10.7)	401 (9.3)	10 (6.8)	4416 (9.0)	

© 2021 Zhou K et al. *JAMA Network Open.*

2013	17 (10.9)	65 (10.5)	397 (9.2)	12 (8.2)	4403 (9.0)	
2014	17 (10.9)	53 (8.6)	423 (9.8)	17 (11.6)	4368 (8.9)	
**Diagnostic confirmation**						.01
Missing	0	0	3	0	24	
Positive histology	133 (85.3)	496 (80.4)	3448 (80.3)	127 (86.4)	40151 (82.2)	
Positive cytology	23 (14.7)	121 (19.6)	844 (19.7)	20 (13.6)	8711 (17.8)	
**Facility Type**						<.001
Missing	1	1	27	0	156	
Non-A/R Program	80 (51.6)	364 (59.1)	2668 (62.5)	132 (89.8)	36930 (75.8)	
A/R Program	75 (48.4)	252 (40.9)	1600 (37.5)	15 (10.2)	11800 (24.2)	
**Distance to treating facility, mean (SD), mile**	15.9 (45.1)	12.2 (27.6)	13.4 (43.8)	58.4 (119.7)	24.5 (89.4)	<.001
**Lymph nodes, mean (SD)** [d]	15.1 (34.7)	13.4 (32.9)	13.5 (33.0)	14.1 (33.6)	15.0 (34.3)	<.001
**Charlson-Deyo Score**						<.001
0	98 (62.8)	433 (70.2)	2361 (55.0)	75 (51.0)	27598 (56.5)	
1	39 (25.0)	123 (19.9)	1228 (28.6)	50 (34.0)	14660 (30.0)	
2	14 (9.0)	46 (7.5)	505 (11.8)	14 (9.5)	5023 (10.3)	
>=3	5 (3.2)	15 (2.4)	201 (4.7)	8 (5.4)	1605 (3.3)	
**Surgery of Primary Site**						.11
Missing	0	0	12	0	145	
No	153 (98.1)	604 (97.9)	4195 (97.9)	144 (98.0)	47425 (97.3)	
Yes	3 (1.9)	13 (2.1)	88 (2.1)	3 (2.0)	1316 (2.7)	
**Radiation Therapy**						.40
Missing	0	8	26	2	279	
No	65 (41.7)	208 (34.2)	1473 (34.5)	54 (37.2)	16765 (34.5)	
Yes	91 (58.3)	401 (65.8)	2796 (65.5)	91 (62.8)	31842 (65.5)	
**Chemotherapy**						.41
Missing	6	7	69	4	706	
No	23 (15.3)	105 (17.2)	681 (16.1)	18 (12.6)	8153 (16.9)	
Yes	127 (84.7)	505 (82.8)	3545 (83.9)	125 (87.4)	40027 (83.1)	
**Median Survival (95CI), mo**	13.4(10.3-16.9)	13.9(12.6-15.2)	13.6(13.2-14.1)	13.5(11.3-16.9)	12.7(12.6-12.9)	<.001

AA, African American; NA, Native American; SCLC, small cell lung cancer; A/R, Academic/Research

[a] Reported as No. (%) unless otherwise indicated;

[b, c] Variables refer to the residential region, rather than individual;

[d] Reginal lymph nodes

© 2021 Zhou K et al. *JAMA Network Open.*
